# 

**Published:** 1855-10-01

**Authors:** 


The conclusion of the article on *Oinomania* is unavoidably postponed until our next number.

In the number for January, 1856, will be published, No. 1 of a Series of Papers entitled

**Notes of a Visit**

TO THE

PUBLIC LUNATIC ASYLUMS OF SCOTLAND,

By JOHN WEBSTER, M.D.;

AND

**An Elaborate Essay**

ON SOME

UNRECOGNISED FORMS OF MENTAL DISORDER,

By FORBES WINSLOW, M.D.

### INDEX TO VOL. VIII.

Alcohol, pathological influence of, on the brain, 180
American institutions for the insane, 152, 433, 562
American psychological literature, 375
——— state schools for idiots, 291
Asylums, amusements in, 349
*Asylum Journal*, the, 69
Asylums, organization of, 375
——— state of, in Ireland, 581
Autobiography of the insane, 338
Autopsies performed in the Vienna Lunatic Asylum, 558

Berkeley, psychology of, 354
Bethlem Hospital, annual report of, 4
Brain, the, in relation to the mind, 317
——— researches into the functions of, 512
——— gravity of, in cases of insanity, 573
British asylums for the insane, 1
Bucknill, J. C., M.D., on unsoundness of mind in relation to criminal acts, 207
Buranelli, case of, medico-legally considered, by Forbes Winslow, M.D., D.C.L., 459

Campbell, Dr., on the cause and treatment of insanity, 585
Cartesian psychology, 54, 59
Cerebral pathology, 161
——— symptoms, effects of, on different diseases, 405
Certificates necessary for detention of a lunatic, 121
Colney Hatch Asylum, *post-mortem* examination in, 4
——— third annual report of, 3
Commissioners in Lunacy, their report on seclusion in the treatment of the insane, 30
Consciousness, its different phases, 320
Counsel, conduct of, towards witnesses, 106
Cretinism, researches in, 74
Crime and insanity, tabular statement of, 132
Criminal cases, commissions *de lunatico inquirendo* in, 229
Criminal law, monomania in relation to the, 75
——— anomalous state of, in regard to criminal lunatics, 464
——— responsibility of madmen, 541

Deaf and dumb, case of a gentleman who was born, and subsequently became insane, 591
*De lunatico inquirendo*, evidence in cases, 96
Delasianre, M., on epilepsy, 262
Delirium, considered, 401
*Delirium tremens*, 186
Delusion, non-existence of, no proof of absence of insanity, 118
——— the product of a diseased intellect, 119
Dementia, phenominology of, 402
Demoniacal possession and insanity, 391, 483
Denham, Rev. I. F., on the connexion between morbid, physical, and religious phenomena, 148, 415
Derby County Lunatic Asylum, annual report of, 8
Descartes compared with Bacon, 65
——— his early philosophical studies, 55
——— his theory of substance, 63
——— on psychology, 54
——— on the Divine agency, 65
Devon, County Lunatic Asylum, 15
Disease, moral and intellectual, 216
Divine agency, Descartes on the, 65
Dorset County Lunatic Asylum, annual report of, 20
Drunken madmen, treatment of, 205
Drunkenness, paroxysmal, 193
——— phenomena of, 177
——— writers on, 176, 181
Dundee Asylum, 29
Dyce Sombre *v.* Troup, 447

Earll, Dr. Pliny, on American institutions for the insane, 152
Edinburgh Asylum, 26
Education, influence of, on the mind, 240
Epilepsy and the allied affections, by C. B. Radcliffe, 35
——— cases of, 259
——— hereditary character of, 264
——— how distinguished from other affections, 253
——— its classification as a disease, 49
——— prognosis of, 253
——— statistics of, 263
——— consequences of, 74
——— influences governing the phenomena of, 48
——— the phenomena of, 37
——— varieties of, 41
——— pathology of, 41
——— treatment of, 256, 265
Epileptic tendency, conditions favourable or conducive to the development of the, 45
Ether and chloroform, influence of, on the mind, 589

French psychological literature, 72
Friend's Retreat, the, 12

Galt, Dr. J. M., on pledges extracted from the insane, 134
Gauster, Dr., on the morbid anatomy of the brain, 558
Glasgow Lunatic Asylum, 24
Guislain on insanity, 401

Hallucination, considered, 244
Hanwell Lunatic Asylum, ninth report of, 1
——— ——— report of the matron of, 3
Hawkes, J., on the materialism of insanity, 313
Heigham Hall Lunatic Asylum, Norwich, 168
Herpin, Th., on epilepsy, 35
Homicidal cases of insanity, liberation, of in Ireland, after recovery, 467
Hospitals for the intemperate, 170
Hyperphreny, 248

Idiocy in Ireland, opening of an institution for the treatment of, 587
Idiots, American state school for, 291
——— and lunatics in Ireland, their condition, 328
Insane, American institutions for the, 152, 433
——— Autobiography of the, 338
Insane, British asylums for the, 1
——— imperfect responsibility of the, 209, 222
——— pledges extracted from the, by Dr. J. M. Galt, 134
——— results of experiments on the blood of the, 87
——— Scotch institutions for the, 23
——— seclusion in the treatment of the, report of the Commissioners in Lunacy on, 30
——— alteration of the blood-corpuscles in connexion with physical disease in the, 88, 90
——— the appearance of their blood, 83
——— the histology of their blood, 78
——— great mental impairment in the, without alteration of the blood-corpuscles, 93
——— their aversion to operations, 82
——— their treatment, amelioration of, 348
Insanity and crime, tabular statement of, 132
——— causes of, 11
——— constructive, 218
——— critical remarks on the plea of, by Richard Poole, M.D., 266
——— definitions of, 111, 118, 210
——— hereditary, 13, 202
——— importance of early treatment of, 13
——— its effect on marriage, 95
——— materialism of, 313
——— medico-legal evidence in cases of, 94
——— mode of manifesting, 245
——— modes of establishing the existence of, 111, 127
——— moral, 220
——— origin of, by L. F. E. Renaudin, 233
——— Plea of, in criminal cases, defence of, 128
——— produced by religious excitement, 10
——— symptoms of, 124, 127
——— treatment of, 11, 35
——— use of the stomach-pump in cases of, 13
Instinctive insanity, 217
Intoxicating drinks, adulteration of, 182
Intoxication, crimes produced by, 185
Intemperance, madness produced by, 187
——— ——— the mental pathology of, 175
Intemperate, hospitals for the, 170
Ireland, lunatics and idiots in, statistics of, 331

Ireland, lunatics and idiots in, their condition, 328
Isolation, effects of, on the mind, 252

Jurisprudence, medico-legal, 296
Jury, functions of, 232

Laycock, Dr. Thomas, on the functions of the brain, 512
Legal definition of insanity, 212
——— medicine, 76
Lettsomian lectures delivered before the Medical Society of London, by Forbes Winslow, M.D., D.C.L., 94
Lindsay, W. Lauder, M.D., on the histology of the blood of the insane, 78
Lucid interval, evidence in cases of, 95
Lunacy, how considered by the Roman law, 100
——— the non-restraint system in the treatment of, 71
——— the restraint system in the treatment of, 71
Lunatics and idiots in Ireland, statistics of, 331
——— ——— condition of, 328
Lunatics, proceedings necessary for confinement of, 121
——— moral treatment of, 6

Madness produced by intemperance, 187
Malebranche, his opinions, 359
Man, connexion of the physical and somatic element of, 234
Materialism, Berkeley's opinions concerning, 354
——— its signification, 361
Mayo, Thos., M.D., F.R.S., on medical testimony in cases of lunacy, 207
Medical assessors in cases of insanity, 230
——— juries in cases of insanity, 231
——— witnesses, duties of, 100, 103, 120
Medico-legal evidence in cases of insanity, 94
——— jurisprudence, 296
Memory, its functions, 324
Mental alienation, types of, 236, 248, 262
——— diseases, on the causes and morbid anatomy of, by John Webster, M.D., F.R.S., 139, 282
——— philosophy, on the uses and influence of, by M. J. Rae, M.D., 379
Mind, development of its faculties, 324
——— on the, 408
——— the, in relation to the brain, 317
Mind, study of the philosophy of, 493
Monomania, nosology of, considered, 249
——— in relation to the criminal law, 75
Morningside Asylum, narrative of a residence in, 338

Natural capacity considered, 327
Non-restraint system, 6, 10, 30, 71
——— ——— originator of the, 72
Norfolk County Asylum, annual report of, 23
——— Lunatic Asylum, annual report of, 8
North and East Riding of York Asylum, management of, 71

Oinomania, 175, 199, 202
Opium-eater, confessions of an, 183, 189
——— its effects on the mental system, 183, 189

Paralysis, general, 404, 409
Parliamentary report of the number and condition of lunatics and idiots in Ireland, 328
Physical and religious phenomena, morbid connexion between, 415
Pinel on the humane treatment of the insane, 556
Plea of insanity, critical remarks on, 466, 421
Polydipsia ebriosa, 176
Poole, Richard, M.D., on the plea of insanity, 466, 421
Psychological investigations, importance of, 381
——— ——— prejudice against, 379
Psychological literature, American, 375
——— ——— French, 72
Psychology of Berkeley, 354
Psychological terminology, a new, suggestions for, 173
Puerperal mania, treatment of, 309

Radcliffe, C. B., M.D., on epilepsy and the allied affections, 35
Rainhill Asylum, annual report of, 23
Ray, M. J., M.D., on the uses and influence of mental philosophy, 379
Religious excitement in producing insanity, 10
——— consolation to the insane, 583
——— instruction of the insane, 463
——— persuasions, statistics of, 472
——— phenomena, 478
Renardin, L. F. E., on the origin of insanity, 233
Restraint in cases of lunacy, 71

Scotch institutions for the insane, 23
Seclusion in the treatment of the insane, 30, 32
Skae, Dr., on cerebral pathology and specific gravity of the brain in cases of insanity, 573
Softening, cerebral, 409
Somerset County Asylum, annual report of, 22
Souter, Rev. Jos., on demoniacal possession, 391
——— religious consolation to the insane, 583
Staffordshire Lunatic Asylum, annual report of, 22
St. Luke's Hospital, annual report of, 22
Stephen on the criminal responsibility of the insane, 541
Suffolk Lunatic Asylum, annual report of, 17
Suicidal mania in the drunkard, 188
Surrey Lunatic Asylum, annual report of, 7
Swan, Joseph, on the brain in relation to the mind, 322

Testamentary disposition, validity of, 94
Treatment of the insane, claim of priority in the reformation of, 556
——— and causes of insanity, 585
Tuke, Dr. W., a memoir of, 507
Trials, medico-judicial, 378

Unsoundness of mind in relation to criminal acts, by J. C. Bucknill, M.D., 207
——— in its mental and legal considerations, by J. W. Williams, M.D., 207
"Unsound mind," legal meaning of, 96

Webster, John, M.D., F.R.S., on the causes and morbid anatomy of mental diseases, 139, 282
Will made by an insane person, 94
Wilts County Asylum, annual report of, 12
Winn, J. M., M.D., on the treatment of puerperal mania, 309
Winslow, Forbes, M.D., D.C.L., Lettsomian lectures delivered before the Medical Society of London by, 94
Winslow, Forbes, M.D., D.C.L., on the case of Buranelli, 459

York Retreat, the founder of, 507

